# Clinical and biochemical data of endothelial function in Women Consuming Combined Contraceptives

**DOI:** 10.1016/j.dib.2017.05.019

**Published:** 2017-05-12

**Authors:** Irina I. Lobysheva, Sandrine van Eeckhoudt, Flavia Dei Zotti, Ahmad Rifahi, Lucie Pothen, Christophe Beauloye, Jean-Luc Balligand

**Affiliations:** a Institut de Recherche Experimentale et Clinique (IREC), Pole of Pharmacology and Therapeutics (FATH), Université Catholique de Louvain, Brussels, Belgium; b Pole of Cardiovascular Research (CARD), and Departments of Internal Medicine and Cardiovascular Diseases, Cliniques Universitaires Saint-Luc, Université Catholique de Louvain, Brussels, Belgium

**Keywords:** Endothelial function, Vascular oxidative status, Contraceptives, Heme-nitrosylated hemoglobin, Electron paramagnetic resonance

## Abstract

The data presented in this article are associated with the research article entitled “Heme-Nitrosylated Hemoglobin and Oxidative Stress in Women Consuming Combined Contraceptives. Clinical Application of the EPR Spectroscopy” (Lobysheva et al., 2017 [1]), and describe the characteristics of redox status in blood, as well as biochemical and clinical parameters of young female subjects consuming (or not) contraceptive pills (CP). Erythrocyte concentration of reduced thiols reflecting erythrocyte redox capacity was measured before and after sample deproteinization by electron paramagnetic resonance spectroscopy (EPR) using a nitroxide biradical spin probe specifically interacting with reduced thiols; additional data were obtained by a colorimetric method using Ellman׳s reagents in the same samples. The products of nitric oxide oxidation, nitrite and total NO_x_ (in presence of nitrate reductase) were measured in the plasma of study subjects by a colorimetric assay based on the detection of red-violet colored azo dye after reaction of nitrite with the Griess reagent. Biochemical and clinical parameters reflective of cardiovascular risk factors (diastolic blood pressure, C-reactive protein, triglycerides and homocysteine concentrations in venous blood) were compared in subgroups of consumers of CP containing ethinyl estradiol and different types of synthetic progestogens. Parameters reflective of the integrity of the vasculature, - erythrocyte concentration of heme-nitrosylated hemoglobin (5-coordinate α-heme-Fe^II^-NO, HbNO) measured directly by the EPR subtraction method; index of reactive hyperemia response (FRHI) measured by digital pulse tonometry using EndoPAT; oxidative vascular stress measured as total plasma peroxide concentration were compared in subgroups of young women taking CP containing ethinyl estradiol at different concentrations and for various durations.

**Specifications Table**

**Table:** 

Subject area	*Biology*
More specific subject area	*Biomedical science, Endothelial function*
Type of data	*Figures, Text file*
How data was acquired	*Redox status in human erythrocytes and plasma was assayed using EPR spectroscopy (Bruker X-band EPR spectrometer, EMX-micro) and colorimetric assays using SpectraMax i3 (Molecular Devices, LLS, USA); integral endothelial function was assayed by Microplethysmography using peripheral arterial tonometer (Endo-PAT, Itamar, IL)*
Data format	*Raw, Analyzed*
Experimental factors	*Erythrocytes and plasma were separated immediately after venous blood collection from female participants and were processed for biochemical assays and/or stored at -80°C for subsequent measurements by EPR spectroscopy of HbNO and reduced thiols, or peroxide assay*
Experimental features	*Isolated erythrocytes of young female participants consuming or not contraceptive pills were analyzed for the contents of heme-nitrosylated hemoglobin by EPR and of the small molecules containing reduced thiol groups using EPR spin probe and Ellman׳s reagents; plasma of participants was analyzed for the content of nitric oxide metabolites and total biological peroxides.*
Data source location	*Institut de Recherche Experimentale et Clinique; Cliniques Universitaires Saint-Luc, Université Catholique de Louvain; Bruxelles; Belgium*
Data accessibility	*Data are presented in the article*

**Value of the data**

• The data compare the quantitative detection of reduced thiol by electron paramagnetic resonance spectroscopy (EPR) in blood samples using a spin probe and a well-characterized colorimetric method using Ellman׳s reagent, highlighting a new method for precise detection of redox status in erythrocytes applicable to multiple clinical conditions.
• The data provide concentrations of total NOx (nitrite+nitrate) in plasma of female subjects consuming or not contraceptive pills and, by comparison with other techniques, allow to evaluate the accuracy (or lack thereof) of this method for the assessment of the activity of the NO synthase pathway in clinical studies.
• Comparative measurements of diastolic blood pressure, C-reactive protein, triglycerides, and homocysteine concentrations in control female subjects and subjects consuming CP containing ethinyl estradiol and different types of synthetic progestogens can indicate differential levels of cardiovascular risk associated with these components of CP.
• Measurements of erythrocyte HbNO and plasma peroxides combined with endothelial function (FRHI) in female subjects consuming (or not) CP containing ethinyl estradiol at different concentrations and/or for different durations can help to predict CP cardiovascular adverse effects.

## 1 Data

The data describe the effects of CP consumption on cardiovascular risk factors to complete the results presented in [1].

**Fig. 1:**
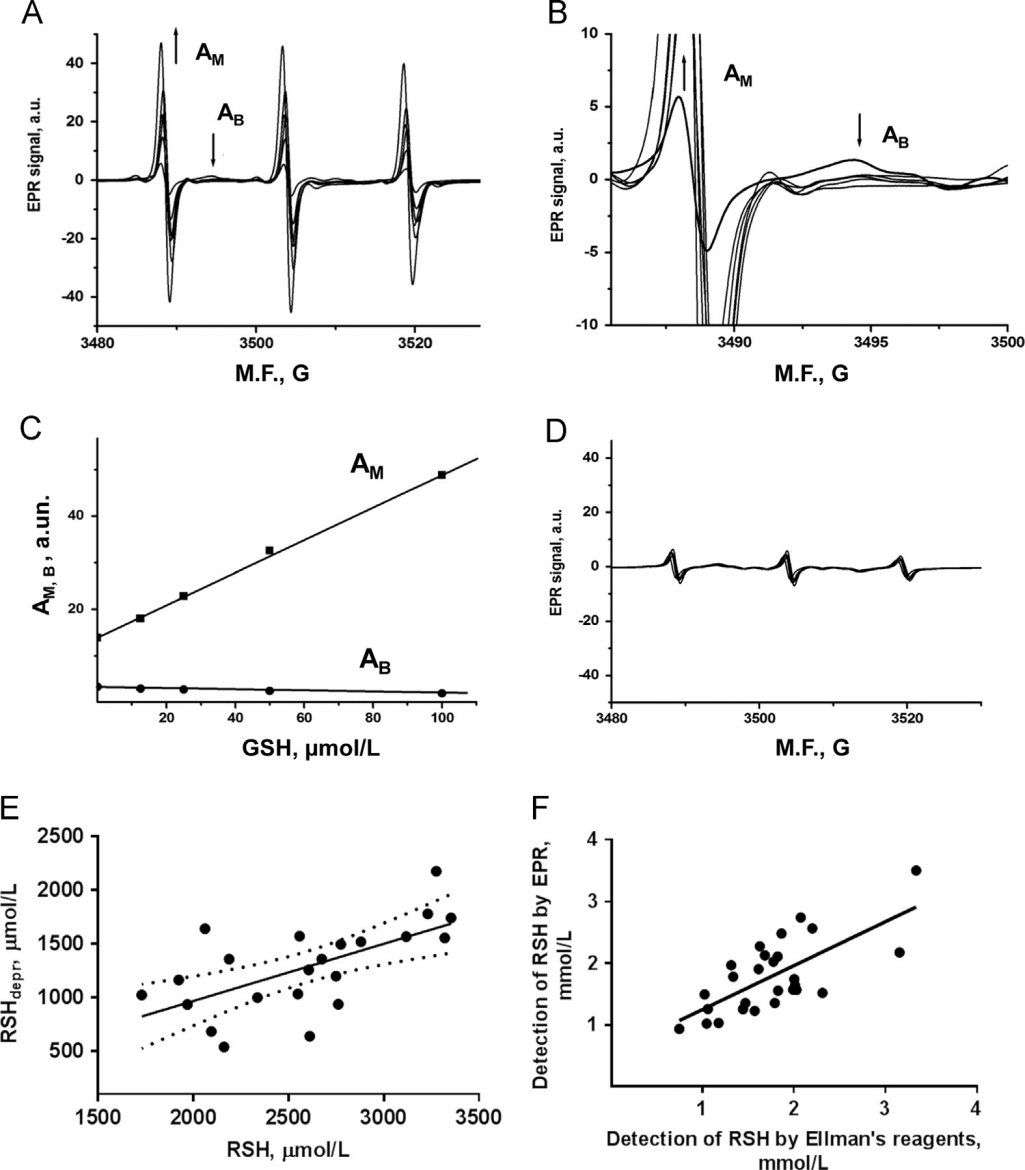
Measurement of reduced thiol concentrations. A. Typical EPR spectra and the low-field spectrum components (B) of the biradical spin probe (200 µmol/L) in presence of increased GSH concentrations; A_M_ is a signal component associated with monoradical nitroxide, A_B_ - with biradical [1]. C. Calibration curve for the quantification of reduced thiol concentrations from the EPR spectra using peak-to-peak amplitude of the A_M_ component (R=0.99; *P*<0.001; *n*=5). D. EPR spectra of the biradical nitroxide recorded in erythrocytes after addition of a SH-group blocker (N-Ethylmaleimide, 3 mM). E. Concentrations of reduced thiols in deproteinized RBCs versus RSH concentrations before deproteinization (*R*^2^=0.4, *P*=0.002; *n*=22). **F**. RSH concentrations in deproteinized RBCs measured by EPR versus the colorimetric assay using Ellman׳s reagent (*R*^2^=0.5; *P*<0.0001; *n*=27).

1.1. A calibration curve for reduced thiol (RSH) quantification by EPR spectroscopy was obtained from the peak-to-peak amplitude of corresponding EPR signals (Fig. 1A–C); the effect of the thiol blocker, N-Ethylmaleimide, is shown in Fig. 1D. The correlation between the RSH concentrations in erythrocyte fractions before and after deproteinization is shown in Fig. 1E (*R*^2^=0.4, *P*=0.002); mean RSH concentration was decreased (by ~36%) after deproteinization. The mean RSH concentrations measured in deproteinized samples by a colorimetric assay using Ellman׳s reagent and by EPR were 1.75±0.11 mmol/L and 1.78±0.11 mmol/L respectively (*n*=27); a linear correlation between measurements by two methods is shown in Fig. 1F (*R*^2^=0.5; *P*<0.0001).

**Fig. 2:**
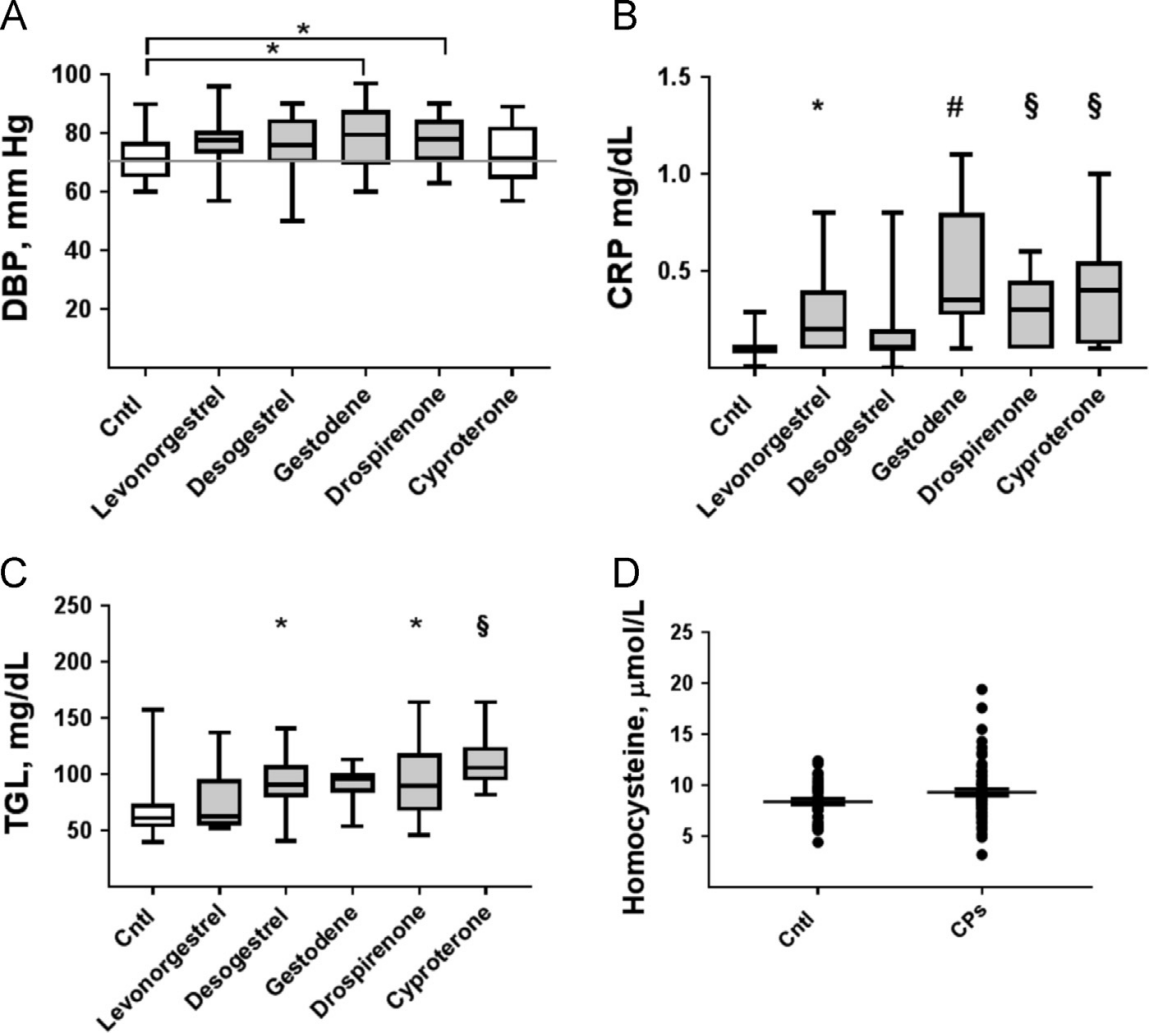
Clinical and biological characteristics of the study cohort. A. Diastolic arterial blood pressure (DBP); B. levels of C-reactive protein (CRP) and C. triglycerides (TGL) in control (Cntl) subjects and users of CP containing ethinyl estradiol and different types of progestogens: levonorgestrel (*n*=11), desogestrel (*n*=15), gestodene *(n*=10), drospirenone (*n*=17) and cyproterone (*n*=8). D. Levels of homocysteine in Cntl subjects and CP users (*n*=43, and *n*=71, respectively); * *P*<0.05; ^§^*P*<0.01; ^#^*P*<0.0001 compared by *t*-test or one-way ANOVA with Bonferroni׳s (A); or Dunn׳s (B and C) corrections for multiple comparisons to Cntl (no significant difference between different progestins).

1.2. The mean values of diastolic arterial blood pressure, plasma concentrations of C-reactive protein and triglycerides of control subjects and consumers of CP containing ethinyl estradiol and different progestins are presented in Fig. 2A–C; the plasma concentrations of homocysteine are shown in Fig. 2D.

**Fig. 3:**
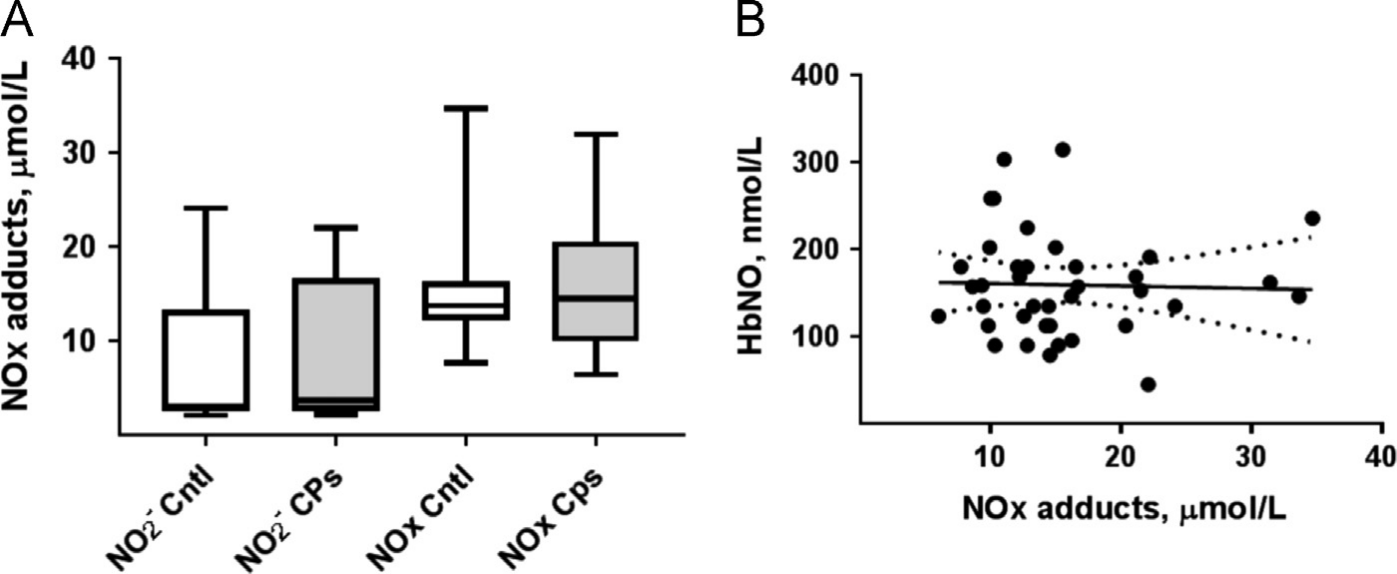
A. Concentrations of the nitric oxide oxidation products (nitrite and NOx) in plasma of control subjects and CP consumers measured by Griess reaction-based assay. Data are presented in box plot (median, 5–95 percentile; *n*=16 and 22 in control and CP groups respectively). B. Erythrocyte concentrations of HbNO measured by the EPR subtraction method versus plasma NOx concentrations in the same subjects (Linear regression analysis: *R*^2^=0.001; *P*=0.8; *n*=38).

1.3. The mean values of the plasma concentrations of the NO metabolites in control subjects and CP consumers measured by an assay based on Griess reaction [2] are shown in Fig. 3A. The correlation between plasma NOx and erythrocyte HbNO concentrations was not significant (Fig. 3B).

**Fig. 4:**
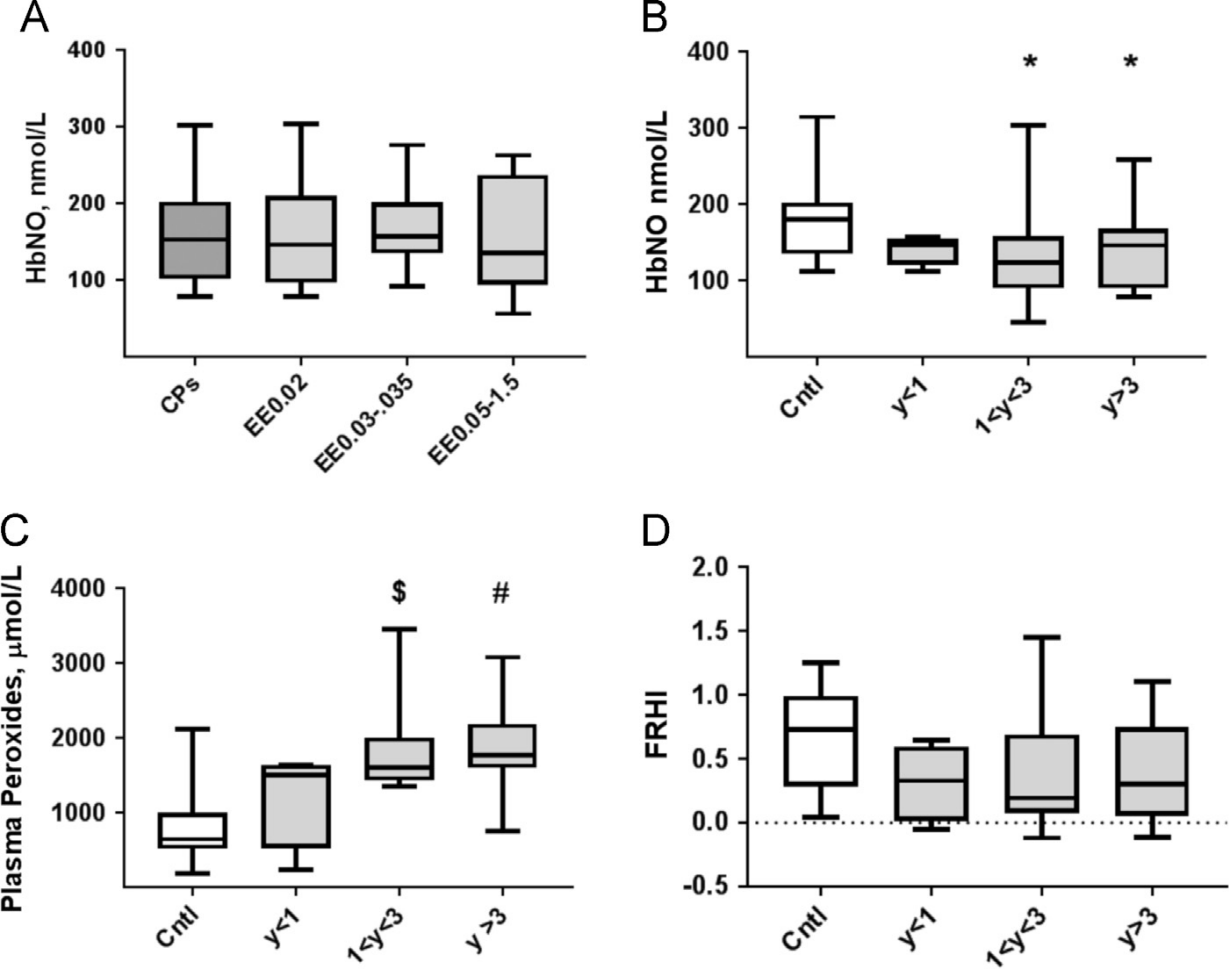
Erythrocyte HbNO, plasma peroxides concentrations and FRHI in users of CP containing different amounts of ethinyl estradiol or after different durations of CP consumption. A. HbNO levels in venous erythrocytes of CP users consuming CPs containing ethinyl estradiol (EE) at different concentrations: 0.02 mg (*n*=37); 0.03–0.035 mg (*n*=17) and 0.05–1.5 mg (*n*=8). B. Erythrocyte HbNO levels; C. plasma levels of biological peroxides; D. indexes of reactive hyperemia response (FRHI) in control subjects and subjects consuming CPs during different periods: *T* < 1; 1 < *T* < 3; *T* > 3 years. * *P* < 0.05; $ *P* < 0.001; # *P* < 0.0001 compared by one-way ANOVA with Dunn׳s correction for multiple comparisons to Cntl.

1.4. The data illustrate the effects of different contents of ethinyl estradiol in CP on circulating HbNO concentrations and of the duration of CP consumption on HbNO, plasma peroxides and endothelial function (FRHI) (Fig. 4A–D).

## 2 Experimental design, materials and methods

### 2.1 Study design

Biochemical and functional tests were performed in female subjects as described in [1] and [3].

### 2.2 Measurements of concentrations of reduced thiols

The concentration of reduced thiols was measured using the EPR probe (*bis*-(2,2,5,5-Tetramethyl-3-imidazoline-1-oxyl-4-yl)disulfide) in deproteinized (or not) erythrocyte samples as described in [1], [4]. Proteins were precipitated by RBC incubation with cold 5% metaphoric acid (v:v=1:5; 15 min) and centrifugation (10,000*g*). Aliquots of supernatants were used for RSH detection by EPR [1] and with Ellman׳s reagent [5]. Briefly, the aliquots were incubated for 5 minutes in Tris buffer (100 mmol/L, pH 8.0) with 0.1 mmol/L DTNB (5,5′-dithiobis(2-nitrobenzoic acid), dissolved in 50 mmol/L of sodium acetate). The absorbance was recorded at 412 nm by a Spectramax i3 (Molecular Devices, LLS, USA). The RSH concentrations were determined from calibration curve with standard solution of N-acetyl-cysteine.

### 2.3 Measurements of nitrite/nitrate, HbNO and biological peroxides

Nitrite and NOx (nitrite plus nitrate) concentrations were measured in plasma samples after ultrafiltration through a filter (PES 10000, VWR), using a Griess reagent kit containing sulfanilamide and N-(1-Naphthyl)ethylenediamine (Enzo Life Sciences, ADI-917-020) following the assay instructions; the nitrite concentrations were determined from a calibration curve (1–100 µM of sodium nitrite). The concentrations of NOx were measured in samples after nitrate reduction by nitrate reductase and NADPH. The concentrations of nitrate were determined from the differential absorption between the samples preloaded or not with nitrate reductase using the calibration curve (1–100 µmol/L of sodium nitrate in presence of nitrate reductase). The absorbance was recorded at 548 nm by Spectramax i3.

HbNO concentrations were quantified from the EPR spectra of erythrocytes recorded on a Bruker X-band EPR spectrometer as described in [1].

Plasma levels of biological peroxides were measured as described in [1].

### 2.4 Statistical analysis

Linear regression analysis, one-way ANOVA with multiple comparisons and t-tests were applied as indicated (GraphPad Prism 7.01).
